# Complementary autophagy inhibition and glucose metabolism with rattle-structured polydopamine@mesoporous silica nanoparticles for augmented low-temperature photothermal therapy and* in vivo* photoacoustic imaging

**DOI:** 10.7150/thno.44668

**Published:** 2020-06-05

**Authors:** Leihou Shao, Yunhao Li, Feifei Huang, Xuan Wang, Jianqing Lu, Fan Jia, Zian Pan, Xinyue Cui, Guanglu Ge, Xiongwei Deng, Yan Wu

**Affiliations:** 1 CAS Key Laboratory for Biomedical Effects of Nanomaterials and Nanosafety, CAS Key Laboratory of Standardization and Measurement for Nanotechnology, CAS Center for Excellence in Nanoscience, National Center for Nanoscience and Technology, Beijing 100190, P. R. China.; 2 Department of General Surgery, Peking Union Medical College Hospital, Peking Union Medical College, Chinese Academy of Medical Sciences, Beijing 100730, P. R. China.; 3 Beijing Key Laboratory of Organic Materials Testing Technology and Quality Evaluation, Beijing Center for Physical and Chemical Analysis, Beijing, 100089, China.; 4 The Key Laboratory of Beijing City on Preparation and Processing of Novel Polymer Materials, Beijing University of Chemical Technology, Beijing, 100029, China.; 5 University of Chinese Academy of Sciences, Beijing 100049, P. R. China.

**Keywords:** autophagy inhibition, rattle structure, polydopamine, glucose oxidase, low-temperature PTT

## Abstract

Rattle-structured nanoparticles with movable cores, porous shells and hollow interiors have shown great effectiveness in drug delivery and cancer theranostics. Targeting autophagy and glucose have provided alternative strategies for cancer intervention therapy. Herein, rattle-structured polydopamine@mesoporous silica nanoparticles were prepared for *in vivo* photoacoustic (PA) imaging and augmented low-temperature photothermal therapy (PTT) via complementary autophagy inhibition and glucose metabolism.

**Methods:** The multifunctional rattle-structured nanoparticles were designed with the nanocore of PDA and the nanoshell of hollow mesoporous silica (PDA@hm) via a four-step process. PDA@hm was then loaded with autophagy inhibitor chloroquine (CQ) and conjugated with glucose consumer glucose oxidase (GOx) (PDA@hm@CQ@GOx), forming a corona-like structure nanoparticle.

**Results:** The CQ and GOx were loaded into the cavity and decorated onto the surface of PDA@hm, respectively. The GOx-mediated tumor starvation strategy would directly suppress the expression of HSP70 and HSP90, resulting in an enhanced low-temperature PTT induced by PDA nanocore. In addition, autophagy inhibition by the released CQ made up for the loss of low-temperature PTT and starvation efficiencies by PTT- and starvation-activated autophagy, realizing augmented therapy efficacy. Furthermore, the PDA nanocore in the PDA@hm@CQ@GOx could be also used for PA imaging.

**Conclusion:** Such a “drugs” loaded rattle-structured nanoparticle could be used for augmented low-temperature PTT through complementarily regulating glucose metabolism and inhibiting autophagy and *in vivo* photoacoustic imaging.

## Introduction

Photothermal therapy (PTT) has been extensively explored as a potent non-invasive strategy for cancer treatment, which utilizes light-absorbing materials to convert photoenergy into local hyperthermia to ablate tumor tissues 1,2. However, common PTT strategy generally needs sufficient heating to attain harsh photothermal temperatures (e.g. > 50 °C) to induce effective cell necrosis, which inevitably requires large amount of photothermal agents or a high-power laser density 3-5. Furthermore, it may also lead to collateral damage to nearby normal tissues and induce inflammatory diseases by the indiscriminate heating. Recently, one emerging strategy to over these limitations could be achieved by mild low-temperature PTT pattern (e.g. < 45 °C). However, the efficacy of low-temperature PTT is restricted and can be repaired by cells themselves through the help of thermal resistance 6-10. The thermal resistance is mainly ascribed to the effects of heat shock proteins (HSPs), which would also lead to tumor recurrence and metastasis 11-13. Therefore, it is a feasible strategy to enhance low-temperature PTT by inhibiting the expression of HSPs. Current reversible strategies for thermal resistance were mainly adopted by introducing various HSP inhibitors, including small molecule inhibitors and small interference RNAs 14-15. Nevertheless, the therapeutic effect by single low-temperature PTT was still restrictive. Therefore, it remains the imperative needs to develop innovate strategy to improve the low-temperature PTT effects.

Cancer starvation therapy is an emerging approach by cutting off the nutrient supply to suppress tumor growth, which is usually achieved via vascular embolization 16-18. Alternatively, in consideration of the sensitivity of tumor cell to glucose, tumor starvation therapy has drawn great interest by consuming the glucose via biocatalysts 19-21. In particular, glucose oxidase (GOx) was the most usually used biocatalyst for tumor glucose-metabolism regulation, which could convert glucose to glucose acid and toxic H_2_O_2_. Specially, the enhanced glucose depletion by GOx would inhibit the ATP production and the expression of HSPs, which would also lead to enhanced PTT effects 9.

Autophagy is a catabolic process maintaining cellular homeostasis through degradation misfolded proteins or damaged organelles by the lysosomal system 22. In normal tissue, autophagy is a highly conservative cellular process, while dysfunctional autophagy acts a crucial role in many diseases, including cancer, microbial infection and neurodegeneration 23,24. Moreover, autophagy is essential for cell survival in response to various cellular stresses. Both metabolic stress and PTT would cause autophagy activation in cancer cells. In addition, emerging studies have revealed that activated autophagy plays an important role in producing resistance against multiple therapeutic approaches 25-29. Thus, combined autophagy inhibition has been regarded as a prevailing route to enlarge the tumor treatment sensitivity, including chemotherapy, radiation or hyperthermal 30,31.

Owing to their outstanding properties, rattle-type nanostructures with movable cores, porous shells, and interstitial hollow spaces in between, have attracted increasing attention in the fields of nanomedicine 32,33. Moreover, the nanoparticles could also be an intelligent platform for multiaspect application by bringing in a multifunctional core. As a neurotransmitter, dopamine was widely distributed in humans 34. Specially, dopamine could self-polymerize to form surface-adherent polydopamine (PDA) nanoparticles or PDA coatings onto a wide range of inorganic materials 35-38. Interestedly, the photothermal effect of PDA nanoparticles has also been widely explored for photoacoustic (PA) imaging and PTT with good biocompability and photothermal stability 39.

**Figure 1:**
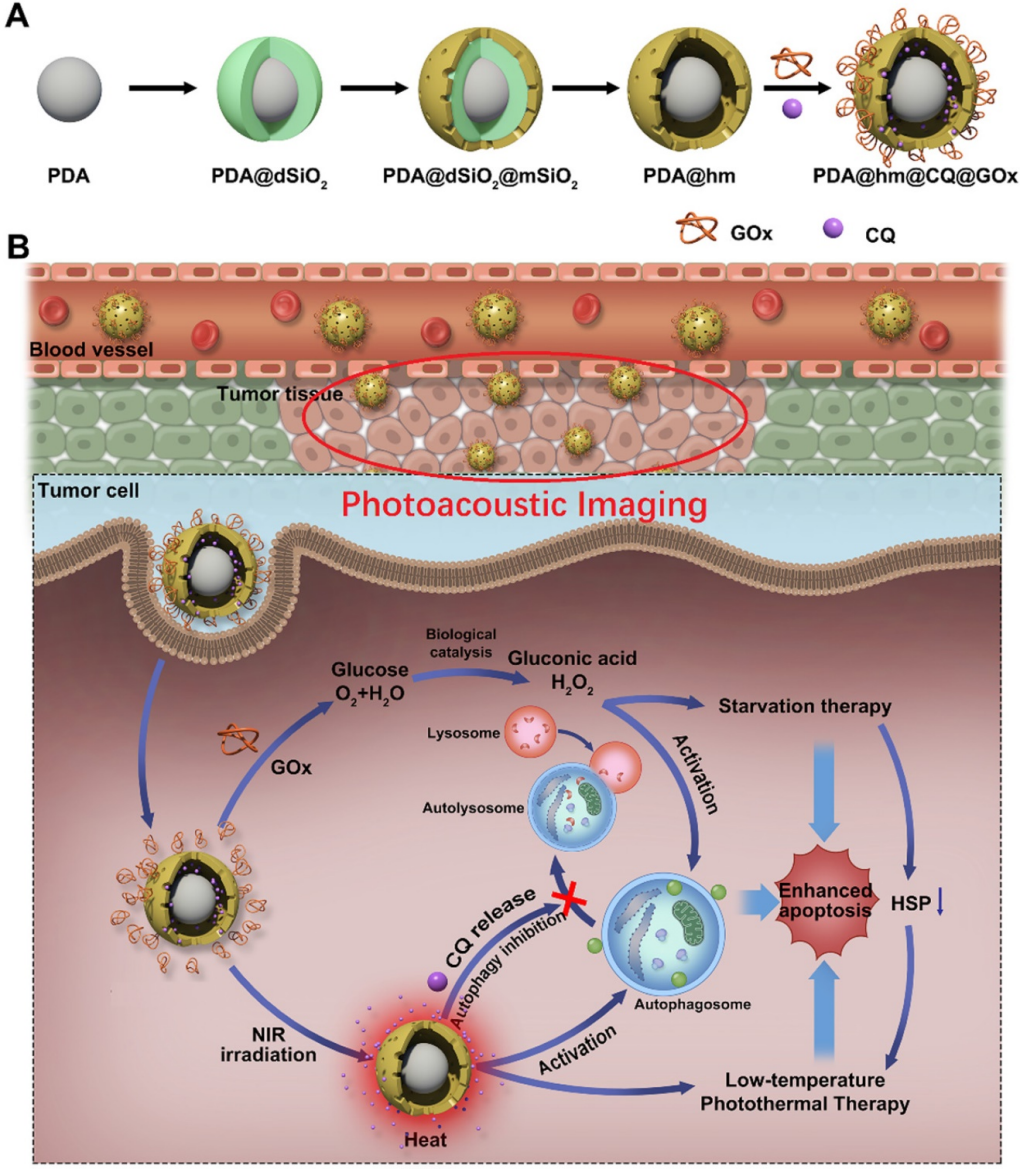
** (A)** Schematic illustration of the multi-step preparation process of the rattle-structured PDA@hm@CQ@GOx nanoparticles. Polydopamine core/hollow mesoporous silica shell nanoparticles (PDA@hm) with a rattle structure was designed and prepared. CQ was introduced to the large cavity of PDA@hm and GOx was decorated onto the surface of PDA@hm by covalent linkage (PDA@hm@CQ@GOx). **(B)** Application of the PDA@hm@CQ@GOx for enhanced low-temperature PTT against cancers under NIR laser irradiation by synchronous autophagy inhibition and energy metabolism.

Inspired by these concerns, a multifunctional rattle-structured nanoparticle was designed with the core of PDA and the shell of hollow mesoporous silica (PDA@hm). The central cavity of PDA@hm could be used to load autophagy inhibitor drug chloroquine (CQ) (PDA@hm@CQ). CQ is the only FDA-approved small molecule autophagy inhibitor available for clinical use 40. The glucose consumer of glucose oxidase (GOx) was concurrently conjugated onto the surface of mesoporous silica shell, forming a corona-like structure (PDA@hm@CQ@GOx) (**Figure 1A**). Thus, a strategy of low-temperature PTT in combination with cell energy metabolism and autophagy inhibition was synchronously proposed in this system (**Figure 1B**). Self-polymerized PDA nanocore from dopamine monomer was utilized as the photothermal conversion agent for low-temperature PTT and photoacoustic (PA) imaging contrast for tumor PA imaging *in vivo*. After entering into cancer cells, the enhanced glucose depletion by GOx could suppress the expression of HSP70 and HSP90, leading to an enhanced low-temperature PTT effect. In addition, inhibition of autophagy by CQ could further enhance the therapeutic effects significantly both *in vitro* and *in vivo* with negligible toxicity. In short, we demonstrated the use of these rattle-structured PDA@hm@CQ@GOx nanoparticles for augmented low-temperature PTT through synchronously regulating glucose metabolism and inhibiting autophagy with tumor PA imaging ability.

## Materials and Methods

### Materials

Dopamine hydrochloride was purchased from J&K Chemical Ltd (Beijing, China). Chloroquine diphosphate was purchased from TCI chemical industry development Co., Ltd (Shanghai, China). CCK-8 kit and indocyanine green (ICG) was purchased from Dojindo Molecular Technologies (Tokyo, Japan). LysoTracker Green was obtained from Molecular Probes Inc. (Eugene, OR). Antibodies were purchased from Abcam, Inc. All other chemicals and solvents were of analytical grade commercially available unless specially mentioned otherwise. Ultrapure water (deionized (DI) water) was supplied by a Milli-Q water system (Millipore, Bedford, MA, USA).

### Preparation of PDA, PDA@dSiO_2_, PDA@dSiO_2_@mSiO_2_ and PDA@hm

The PDA nanoparticles were synthesized according to the reported methods 36. Briefly, 1.2 mL of ammonia aqueous solution was added to a 200 mL of flask containing 45 mL of deionized water and 20 mL of ethanol. The mixed solution was then stirred for 30 min at 30 °C. Then, 250 mg of dopamine hydrochloride dissolved in 5 mL of deionized water was quickly added into the above solution under vigorous stirring and aerobic conditions. After stirring for 24 h, the PDA nanoparticles were obtained by centrifugation and purified by repeated washing with deionized water.

For the synthesis of PDA@dSiO_2_, 2 mL of PDA nanoparticles solution (3 mg/mL) was mixed with 0.628 mL of ammonia aqueous solution and 14.28 mL of ethanol in a 50 mL flask under vigorous stirring at 30 °C for 30 min. Then, 0.106 mL of tetraethyl orthosilicate (TEOS) dissolved in 0.94 mL of ethanol was added dropwise into the above solution. After stirring for 30 min at 30 °C, the PDA@dSiO_2_ nanoparticles were obtained by centrifugation and purified by repeated washing with ethanol.

Hexadecyl trimethyl ammonium bromide (CTAB) was used as the template to modify PDA@dSiO_2_ with mSiO_2_ according to the previous work with some modification 41,42. Typically, 18 mL of deionized water was mixed with 0.8 mL of CTAB (0.2 M) solution and 0.2 mL of NaOH (0.1 M) solution under stirring. 1 mL PDA@dSiO_2_ solution was added into the above mixture and stirred for 30 min at 30 °C. Subsequently, 25 μL of TEOS in 225 μL of ethanol was added dropwise into the above solution. After stirring for 30 min at 30 °C, the CTAB molecules were removed by repeated washing with methanol. Finally, the PDA@dSiO_2_@mSiO_2_ nanoparticles were collected by centrifugation and purified by repeated washing with ethanol, deionized water.

Finally, the intermediate dSiO_2_ layer was selectively etched via a typical “surface-protected selective etching” method 42. 10 mL of PDA@dSiO_2_@mSiO_2_ solution was mixed with 10 mL of polyvinylpyrrolidone (PVP, Mw=40000) solution and the mixed solution was stirred for 30 min at 30 °C. Then, the temperature was raised to 95 °C and maintained for 2.5 h to etch the layer of dSiO_2_. Thereafter, the solution was cooled down to room temperature. The PDA@hm nanoparticles were collected by centrifugation and purified by repeated washing with ethanol and deionized water, respectively.

### Preparation of PDA@hm@CQ@GOx

The CQ was loaded on the hollow cavity of PDA@hm. CQ was suspended in deionized water and then added into PDA@hm solution under stirring in the dark at room temperature. After 36 h, the PDA@hm@CQ products were collected via centrifugation and washed with deionized water three times to remove the unloaded CQ. To make GOx covalently grafted onto the surface of PDA@hm, the PDA@hm nanoparticles were treated with 3-aminopropyltrimethoxysilane (APTES) to form the amino groups on their surface. Then, a certain amount of GOx and EDC, NHS were dissolved in 6 mL ultrapure water and stirred for 8 h at room temperature. The amino-functionalized PDA@hm was added into the above solution and further stirred for 24 h. The final PDA@hm@GOx were collected by centrifugation and purified by ultrapure water washing.

### Characterizations

Morphological characterizations were conducted by using scanning electron microscopy (SEM) (Hitachi S4800) and transmission electron microscopy (TEM) (Tecnai G2 20 S-TWIN, FEI). Size distribution of nanoparticles was recorded by using a Zeta-sizer NanoZS (Malvern, UK). Nitrogen adsorption-desorption isotherms and pore size distribution curves were measured using Micromeritics Tristar II 3020.

### Photothermal evaluation

The photothermal effects of PDA@hm solutions with various concentrations were evaluated by irradiating with an 808 laser at a power density of 1.5 W/cm^2^. The temperature changes were monitored and recorded by an infrared thermal camera.

### Cellular uptake assays

To investigate the cellular uptake behavior of the PDA@hm, fluorescent dye ICG was loaded on the hollow cavity of PDA@hm to track the distribution of nanoparticles in cells. HepG-2 cells were seeded in glass-bottomed dishes for 24 h at 37 °C incubator with 5% CO_2_. Then, the original culture medium was replaced with the medium containing ICG loaded PDA@hm nanoparticles. After another 0.5, 2, or 4 h incubation, the cells were washed with PBS and then the cell nucleus and lysosomes were stained by Hoechst 33342 and LysoTracker Green, respectively. Finally, the cells uptake images were observed by a confocal laser scan microscopy (CLSM, Carl Zeiss).

### Western-blot assays

HepG-2 cells were seeded in a 6-well plate at a density of 1 × 10^5^ cells per well for 24 h and then performed with different operations. After that, the cells in each group were collected and lysed. The proteins were separated on gel and subsequently transferred onto a poly (vinylidene difluoride) (PVDF) membrane. After blocking with 5% (w/v) milk, the membranes were incubated with the primary antibody overnight at 4 °C and then incubated with the secondary antibody for 1 h at room temperature. A ChemiDoc XR + UV illuminator was used to detect the different protein bands.

### *In vitro* cytotoxicity assays

The *in vitro* cytotoxicity assay was carried out by cell counting kit-8 (CCK-8) assay. HepG-2 cells were cultured in 96-well plates with a density of 5×10^3^ cells per well and cultured overnight. After that, the original culture medium was removed and replaced by fresh medium containing various formulations combined with different NIR treatments. After further incubation for 24 h or 48 h, the cells viabilities were measured by CCK-8 assay.

### Apoptosis assay by flow cytometry

Flow cytometry assay was used to evaluate the cells apoptosis rate after different formulations treatments. HepG-2 cells were seeded into 6-well plates and cultured overnight. After treated with PBS, PDA@hm, CQ, GOx, PDA@hm+NIR, PDA@hm@CQ+NIR, PDA@hm@GOx+NIR, PDA@hm@CQ@GOx+NIR, the cells in each well were collected and stained with annexin V-FITC and PI, and then detected by flow cytometry.

### Autophagy inhibition analysis by TEM

HepG-2 cells were incubated with PDA@hm, CQ, GOx, PDA@hm+NIR, PDA@hm@CQ+NIR, PDA@hm@GOx+NIR, and PDA@hm@CQ@GOx+NIR in culture medium, respectively. After that, the cells were harvested and fixed with 2.5% glutaraldehyde in PBS buffer overnight. After washing, the cells were fixed with 1% osmium and dehydrated with a graded series of ethanol. Subsequently, the cells were embedded in epoxy resin and sectioned (70 nm), stained and finally observed under TEM.

### Animals

The animal experiments were conducted following protocols approved by the Experimental Ethics Committee in Beijing. The HepG-2 tumor-bearing mice was generated by the standard subcutaneous inoculation on female BALB/c mice.

### *In vivo* PA imaging

When the tumor volume reached around 150 mm^3^, the mice were intravenously injected with 100 μL of solution containing PDA@hm@CQ@GOx. The *in vivo* imaging was then carried out by a multispectral optoacoustic tomography scanner (MSOT, iThera Medical) at different time points after injection.

### *In vivo* anti-tumor studies

When the tumor volume reached around 100 mm^3^, the HepG-2 tumor bearing mice were randomly divided into 8 groups (saline, PDA@hm, PDA@hm@CQ, PDA@hm@GOx, PDA@hm+NIR, PDA@hm@CQ+NIR, PDA@hm@GOx+NIR, PDA@hm@CQ@GOx+NIR), and then intravenously administrated with corresponding formulations. After 24 h post-injection, the mice treated with NIR treatment were irritated by 808 nm NIR laser for 5 min. The tumor volumes of all groups were measured with a digital caliper and calculated by the following formula: tumor volume = tumor width^2^ × tumor length/2. The body weights of mice were also recorded every other day. After 16 days, all the mice were sacrificed and the tumors and main organs were collected for following histological analysis.

### Serum biochemical analysis

At the end of treatments, mice were sacrificed and serum were collected for blood biochemical analysis to systemically evaluate the biosafety of various nanoparticles (Charles River Laboratories, Beijing, China).

### Statistical analysis

The data were presented as mean ± standard deviation (SD), and the statistical significance was determined on the basis of one-way analysis of variance (ANOVA) followed by two-tailed Student's t test. (*P < 0.05, **P < 0.01 and ***P < 0.001).

## Results and Discussion

### Synthesis and characterization of PDA@hm@CQ@GOx nanoparticles

**Figure 2:**
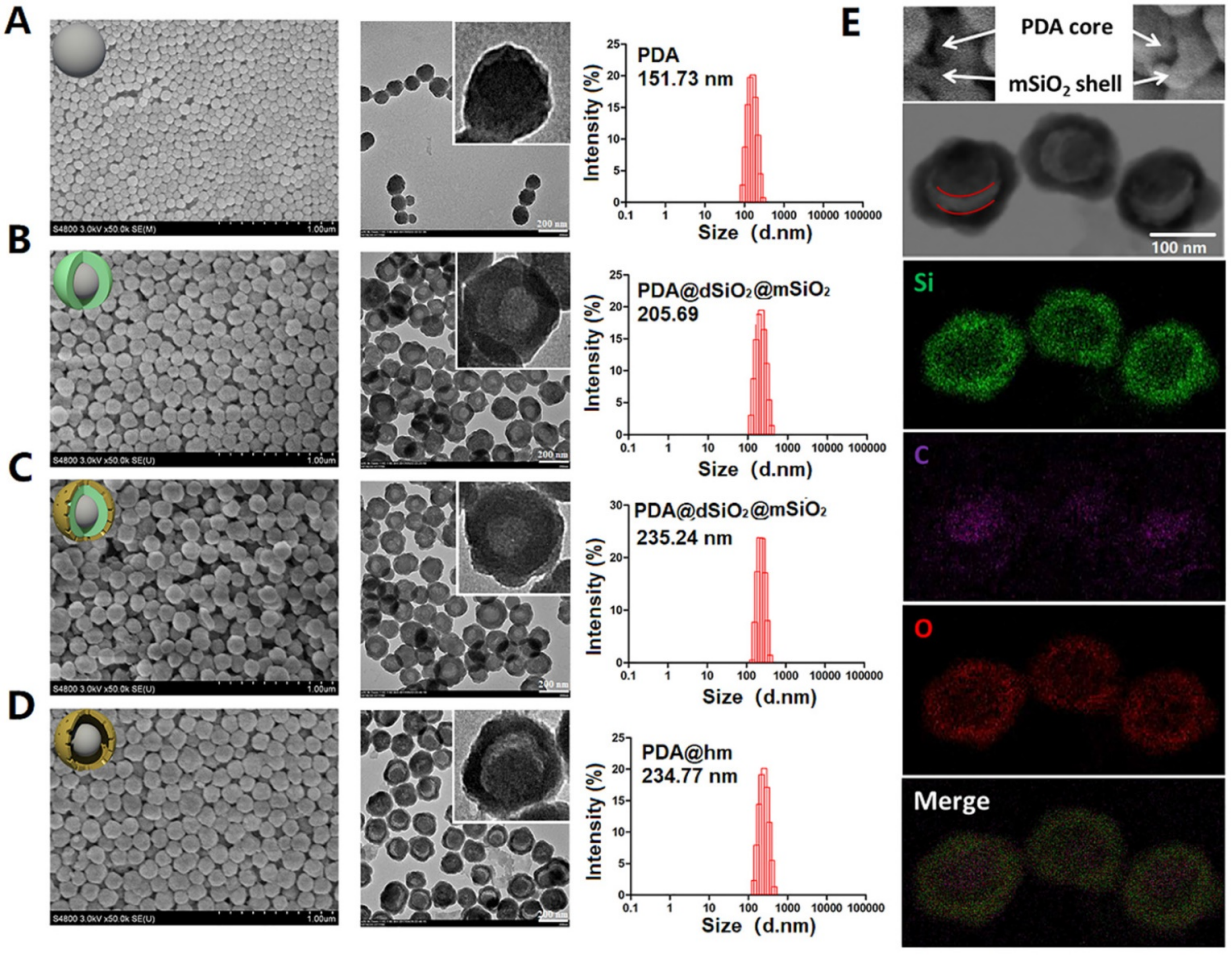
SEM, TEM, DLS and elemental analysis of corresponding nanoparticles. **(A)** PDA, **(B)** PDA@dSiO_2_, **(C)** PDA@dSiO_2_@mSiO_2_, **(D)** PDA@hm. Average sizes of PDA, PDA@dSiO_2_, PDA@dSiO_2_@mSiO_2_ and PDA@hm were 151.73 nm, 205.69 nm, 235.24 nm and 234.77 nm, respectively. **(E)** Corresponding elemental (Si, O, C) mappings of PDA@hm.

The rattle-structured polydopamine core/hollow mesoporous silica shell nanoparticles (PDA@hm) were rationally designed and synthesized by a four-step process depicted in **Figure 1A**. First, PDA nanoparticle was synthesized as the core via self-polymerization of dopamine monomer under alkaline environment with the oxidant of oxygen 36,43. Second, a dense silica layer was coated onto the surface of PDA nanoparticle to fabricate PDA@dSiO_2_ through the hydrolysis of tetraethyl orthosilicate (TEOS). Third, a mesoporous silica shell was further decorated onto the PDA@dSiO_2_ by using hexadecyltrimethylammonium bromide (CTAB) as the template via the hydrolysis of TEOS, forming PDA@dSiO_2_@mSiO_2_ nanoparticles 41,42,44. Finally, hot water was used as the etchant to selectively etch out the intermediate dSiO_2_ layer and polyvinylpyrrolidone (PVP) was chosen as the protecting agent to protect the surface of outer mSiO_2_ shell. An internal cavity between the PDA nanocore and outer mesoporous silica shell was formed via a typical “surface-protected selective etching” method 42,45, generating rattle-structured PDA@hm. The scanning electron microscope (SEM), transmission electron microscope (TEM) and dynamic light scattering (DLS) analysis of PDA, PDA@dSiO_2_, PDA@dSiO_2_@mSiO_2_ and PDA@hm nanoparticles were shown in **Figure 2A-D**, respectively. SEM and TEM results revealed that all the obtained nanoparticles exhibited mono-dispersity and spherical shape. A conspicuous increment in the diameters could be observed after dSiO_2_ layer and mSiO_2_ shell superposition. The mean diameters of PDA nanocore, PDA@dSiO_2_, PDA@dSiO_2_@mSiO_2_ and PDA@hm were calculated to be 151.73 nm, 205.69 nm, 235.24 nm and 234.77 nm from the DLS analysis, respectively. In particular, a typical inner hollow structure between the PDA nanocore and mSiO_2_ shell could be clearly observed from the TEM image of PDA@hm, confirming the successful construction of rattle-structured nanoparticles (**Figure 2E**). The hollow property of PDA@hm could be used to load drugs and the PDA core could be used as photothermal agent for PTT.

The major elements (Si, O, C) displayed in the element mapping images showed that Si and O signals were distributed within the shell and C signal was mainly distributed within the core, also demonstrating the successful formation of PDA core and mSiO_2_ shell in PDA@hm (**Figure 2E**). In addition, the nitrogen adsorption-desorption analysis also confirmed the existence of the hollow structure of PDA@hm. As shown in **Figure S1**, the Brunauer-Emmett-Teller (BET) surface area of PDA@hm was measured to be 169.2 m^2^/g, while the parameter of PDA@dSiO_2_@mSiO_2_ was 129.1 m^2^/g. The increased surface area of PDA@hm could be contributed to the existed hollow cavity in PDA@hm. The average pore diameter of PDA@hm was measured to be 4.9 nm (**Figure S2**). Subsequently, the autophagy inhibitor CQ was loaded into the hollow cavity and GOx was covalently conjugated onto the surface of PDA@hm, respectively. As calculated, the optimal PDA@hm@CQ was prepared at a mass feeding ratio of 1.5:1 (CQ to PDA@hm) and this ratio was selected in our following experiments. The loading capacity of CQ and the conjugation efficacy of GOx in the obtained PDA@hm@CQ@GOx were 15 wt% and 5 wt%, respectively. DLS, SEM and TEM analysis of PDA@hm@CQ@GOx demonstrated that CQ/GOx incorporation could lead to slight increasement in the size of nanoparticles (**Figure S3-S5**). Notably, the GOx after conjugation still maintained its high biocatalytic activity to glucose (**Figure S6**). In addition, the PDA@hm@CQ@GOx exhibited good stability in different solutions after storage in a long time (**Figure S7**).

**Figure 3:**
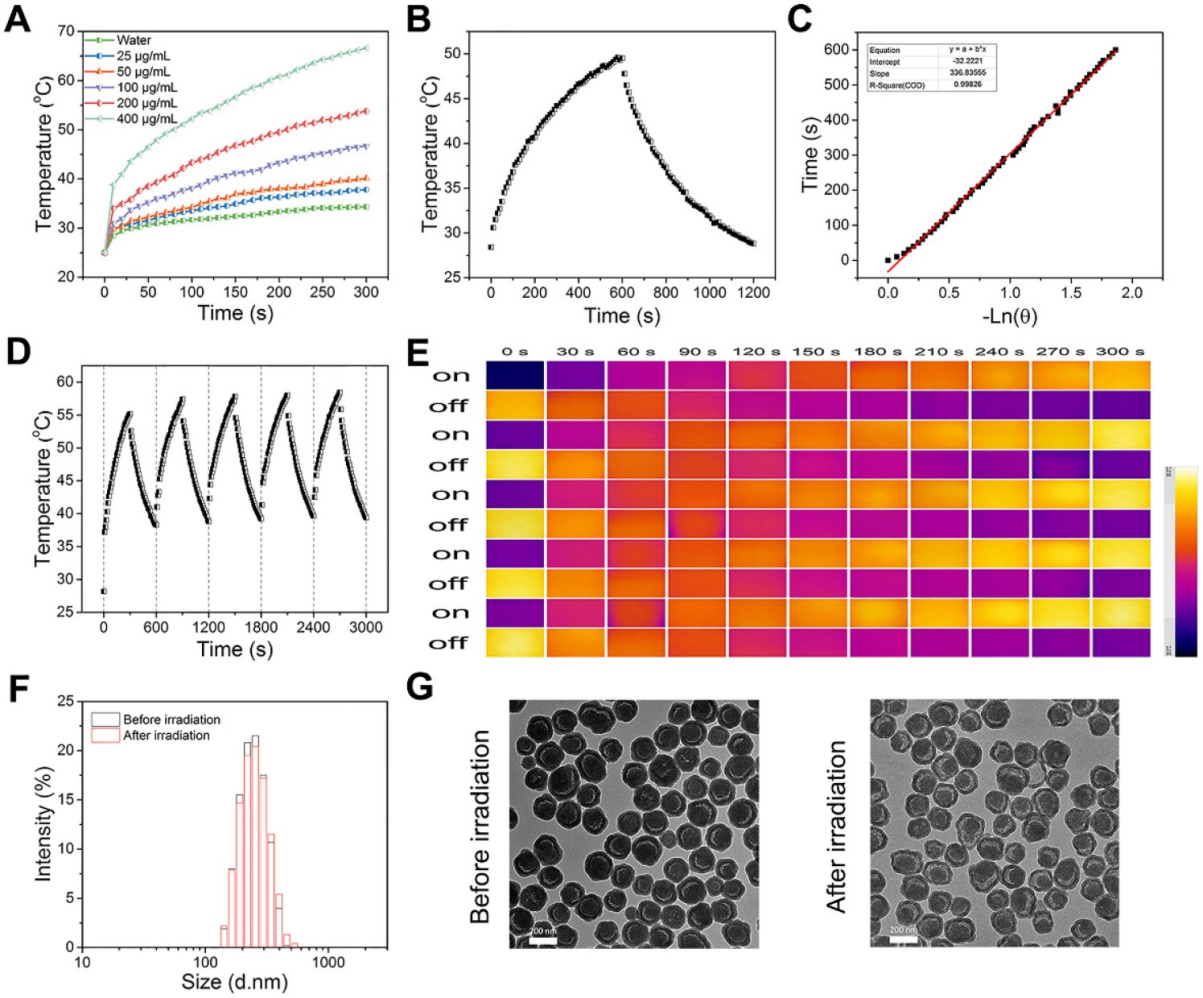
**(A)** Photothermal-heating curves of PDA@hm@CQ@GOx solutions at different concentrations under 808 nm laser irradiation. **(B)** Temperature changes of PDA@hm@CQ@GOx solution under 808 nm laser irradiation for 600 s, and then followed by a cooling period. **(C)** The time constant (τ_s_) for the heat transfer in this system calculated from the linear time data during the cooling period. **(D)** Photothermal stability of PDA@hm@CQ@GOx solution over repeated irradiation on/off cycles. **(E)** The corresponding thermal images of PDA@hm@CQ@GOx solution over repeated irradiation on/off cycles. **(F)** Hydrodynamic diameters of the PDA@hm@CQ@GOx before and after 808 nm laser irradiation measured by using DLS. **(G)** TEM images of the PDA@hm@CQ@GOx before and after 808 nm laser irradiation.

Considering that PDA-based nanoparticles could be utilized as photothermal agent to convert NIR light energy to heat 46-49, the photothermal effect of PDA@hm@CQ@GOx was next systematically investigated with an 808 nm laser at a power density of 1.5 W/cm^2^. The photothermal conversion efficiencies of PDA@hm@CQ@GOx at different concentrations (ranging from 25 to 400 μg/mL) were evaluated under 808 nm laser irradiation for 300 s. As shown in **Figure 3A**, the temperature increasement of PDA@hm@CQ@GOx under laser irradiation exhibited obvious concentration-dependent manner compared with water. When the concentration of PDA@hm@CQ@GOx reached 400 μg/mL, the temperature could increase to 70 °C under continuous irradiation for 300 s whilst the maximal temperature could only increase to about 45 °C with concentration of PDA@hm@CQ@GOx under 100 μg/mL and of 1.5 W/cm^2^ power. In addition, the photothermal conversion efficiency (η) was further measured to assess the photothermal effect of PDA@hm@CQ@GOx (**Figure 3B**). Based on the cooling curve, the η value of PDA@hm@CQ@GOx was calculated to be 30.4 %, which was similar to the η of PDA nanoparticles. The result suggested that PDA@hm@CQ@GOx exhibited excellent thermal conversion capability (**Figure 3C**). It also demonstrated that introduction of mSiO_2_ shell would not affect the photothermal effects of PDA@hm@CQ@GOx nanoparticles compared with bare PDA nanoparticles.

Having verified the photothermal effect of PDA@hm@CQ@GOx, the photostability of PDA@hm@CQ@GOx was further investigated. As shown in **Figure 3D** and **3E**, the temperature elevation curve and the peak temperature of PDA@hm@CQ@GOx did not display significant deterioration during the repeated laser exposure treatment, revealing the excellent photothermal stability and reproducibility of PDA@hm@CQ@GOx. In comparison, ICG was easily photobleached and the photothermal effect dropped obviously after five cycles (**Figure S8**). In addition, TEM and DLS analysis indicated that PDA@hm@CQ@GOx still maintained their morphology and size after 5 irradiation cycles, revealing their high stability (**Figure 3F** and **3G**). Furthermore, the *in vitro* release profiles of CQ from PDA@hm@CQ@GOx indicated that both NIR laser irradiation and mild acidic condition could accelerate CQ release (**Figure S9**).

### *In vitro* therapeutic effects of PDA@hm@CQ@GOx

**Figure 4:**
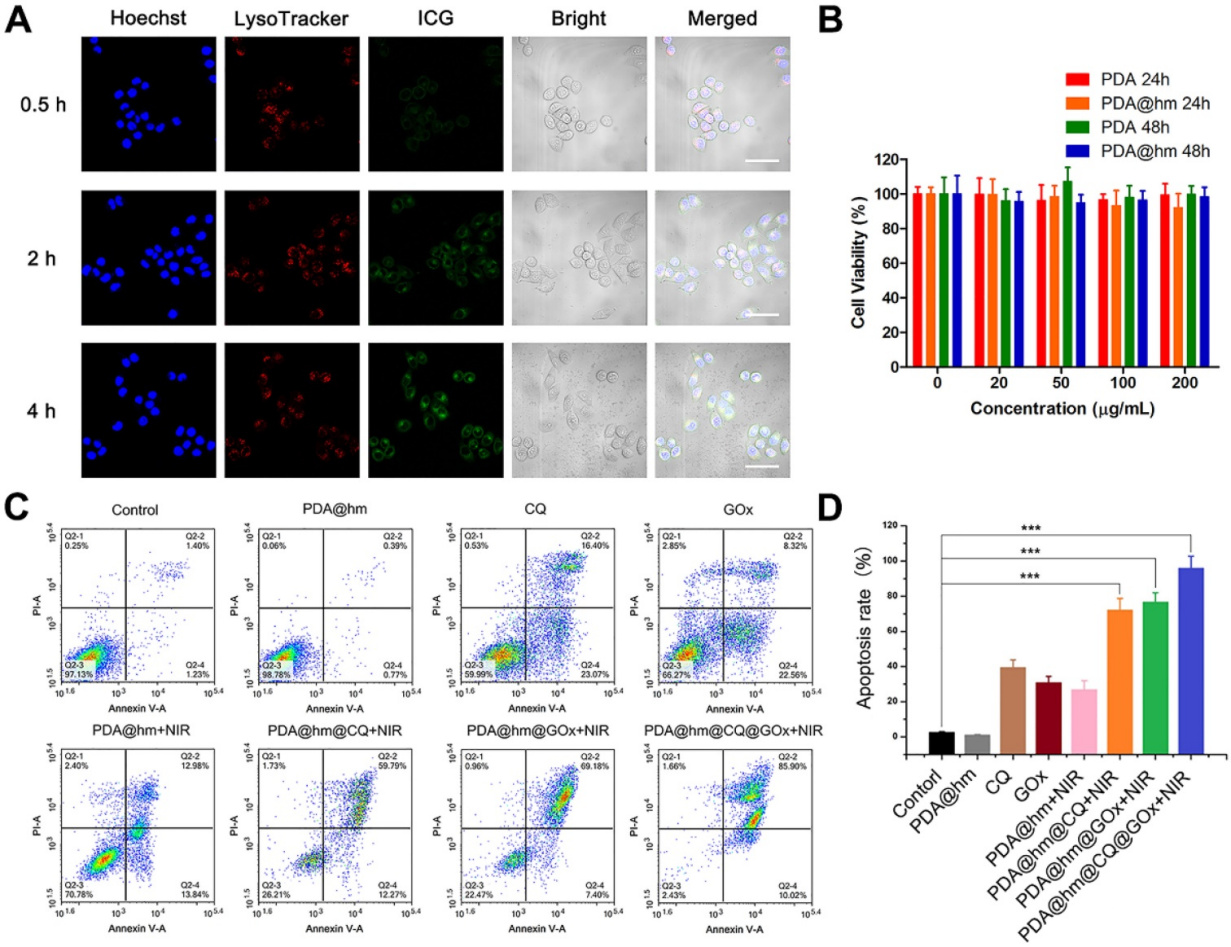
**(A)** CLSM images of the HepG-2 cancer cells after treatment with PDA@hm@ICG@GOx nanoparticles for 0.5 h, 2 h and 4 h. Cells were stained with Hoechst 33342 and LysoTracker Green, respectively (Scale bar: 50 μm). **(B)** Relative viabilities of HepG-2 cells after incubation with PDA or PDA@hm nanoparticles for 24 h or 48 h, respectively. **(C)** Apoptosis rates of HepG-2 cells treated with different formulations determined by flow cytometric analysis. **(D)** Relative apoptosis rates after different formulations treatments. ***p < 0.001.

For cellular uptake studies, indocyanine green (ICG) was used as a model fluorescence molecule loaded into the PDA@hm@ICG@GOx to evaluate the tumor cell uptake behavior by confocal fluorescence microscopy (CLSM). As shown in **Figure 4A**, after co-incubation with PDA@hm@ICG@GOx, the ICG signals could be distinguishable in the HepG-2 cells for 0.5 h. As time extended, the signals became more legible, indicating that PDA@hm@ICG@GOx could be efficiently taken up by HepG-2 cells. Meanwhile, most ICG fluorescence overlapped well with the red fluorescence related to lysosome, demonstrating that the nanoparticles may be internalized by cell endocytosis pathway. The biocompatibility of PDA@hm nanoparticles *in vitro* was therefore evaluated by using HepG-2 cells. As shown in **Figure 4B**, the cell viabilities were still above 90% even at a high concentration of 200 μg/mL, indicating no obvious cytotoxicity was observed in HepG-2 cells after incubation with PDA or PDA@hm nanoparticles for 24 h and 48 h. These results confirmed that the synthesized PDA@hm nanoparticles exhibited good biocompatibility in *in vitro* condition.

We next assessed the therapeutic effects of PDA@hm@CQ@GOx in HepG-2 cancer cells. Herein, we incubated HepG-2 cells with different formulations and then irradiated with 808 nm laser (1.5 W cm^-2^, 5 min) to keep the temperature of cell culture around 45 °C. First, Annexin V-fluorescein isothiocyanate (Annexin V-FITC)/propidium iodide (PI) fluorescence staining was carried out to evaluate the apoptotic and necrotic cells after different treatments by flow cytometry assay. As illustrated in **Figure 4C**, compared with the control, increased cells apoptosis could be observed from the PDA@hm+NIR-, GOx-, and CQ-treated cells, indicating that individual thermal, starvation and autophagy inhibition treatment could induce cell apoptosis. The PDA@hm@CQ+NIR- and PDA@hm@GOx+NIR-treated cells displayed higher apoptosis ratios (72.06% and 76.58%) as compared to single therapy group, indicating the starvation and autophagy inhibition treatment could enhance the pro-apoptotic effect of low-temperature PTT. The highest cell apoptosis ratio reaching almost ~96% was detected in the cells treated with PDA@hm@CQ@GOx+NIR (**Figure 4D**).

**Figure 5:**
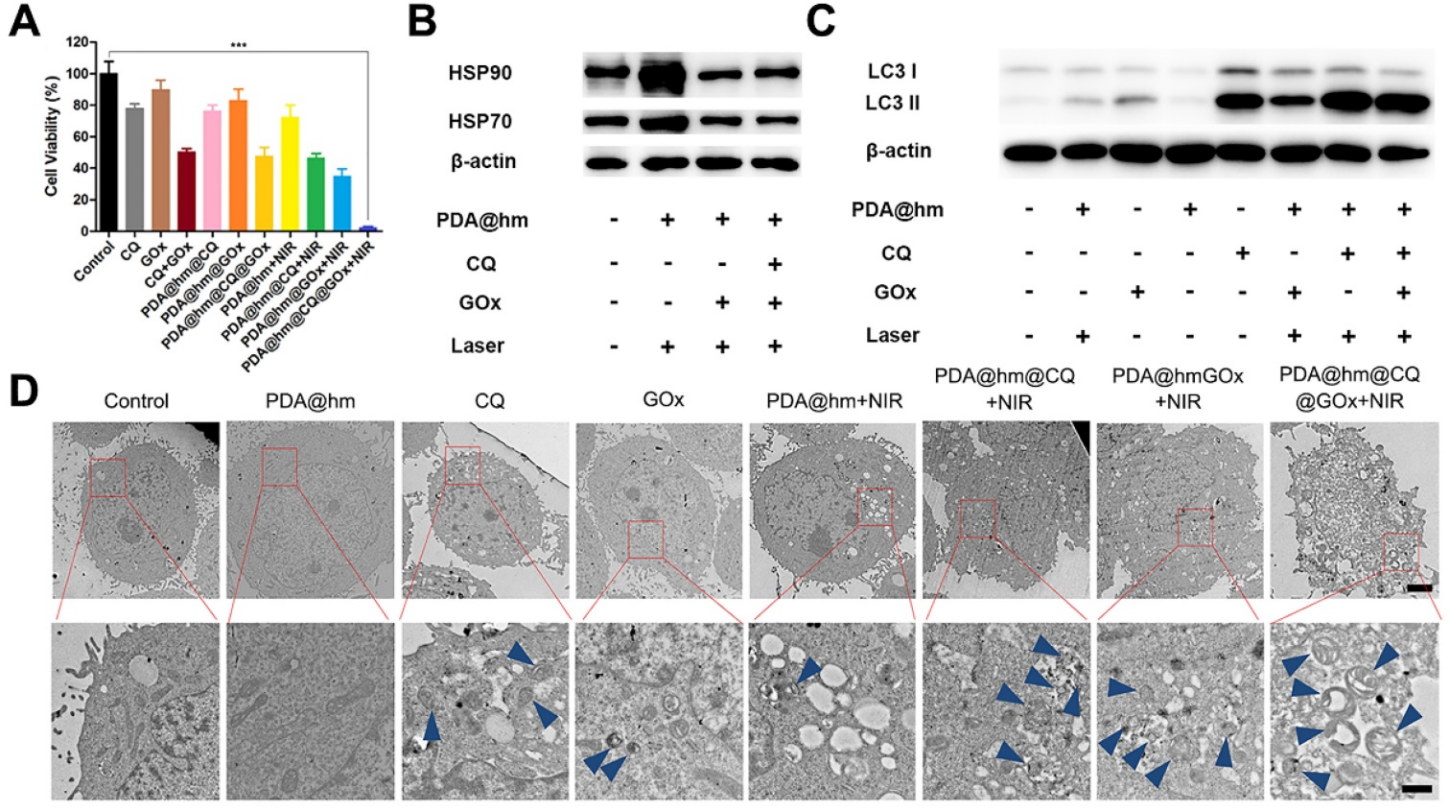
** (A)** Augmented cell cytotoxicity by mild PTT in combination with glucose consumer GOx and autophagy inhibitor CQ in HepG-2 cells. **(B)** Representative expression of HSP90 and HSP70 after different treatments by western-blot analysis. **(C)** Relative LC3-I and LC3-II expression in HepG-2 cells after different treatments. β-actin was used as an internal control. **(D)** Representative TEM images of HepG-2 cells after different treatments. Blue arrows indicated the autophagosome and autolysosome (Scale bar: 2 μm in the upper figure and 500 nm in the enlarged figure). ***p < 0.001.

Furthermore, the cytotoxicity of HepG-2 cells treated with different formulations with or without NIR laser exposure was therefore assessed. As shown in **Figure 5A**, the treatment of CQ, GOx, PDA@hm@CQ and PDA@hm@GOx all displayed a moderate killing effect, implying that individual autophagy inhibition by CQ and glucose depletion by GOx could induce cell death to a certain degree. In comparison, CQ+GOx or PDA@hm@CQ@GOx treatment could lead to a higher cell death rate (nearly 50%), indicated that combining autophagy inhibition and glucose-metabolism regulation resulted in enhanced cells damage effect. PDA@hm upon NIR laser exposure only displayed a moderate killing effect (~75%) due to the limited photothermal effect by low-temperature PTT. By contrast, under equivalent NIR laser exposure, PDA@hm@GOx- or PDA@hm@CQ-treated cells could induce much more cell killing effect than bare CQ or GOx. Of special note, HepG-2 cells treated with PDA@hm@CQ@GOx under NIR laser irradiation displayed the highest cell killing effect nearly 95%. Apoptosis assay together with the cytotoxicity results revealed that mild thermal treatment with synergetic GOx-mediated starvation and CQ-mediated autophagy inhibition by PDA@hm@CQ@GOx+NIR could achieve significant effective PTT performance.

### HSPs suppression and autophagy inhibition induced by PDA@hm@CQ@GOx

We next investigated the related mechanisms based on the above cell apoptosis and cytotoxicity results. As documented in the literature, the glucose depletion by GOx could suppress the expression of HSPs 50. Therefore, the expression of HSP70 and HSP90 in HepG-2 cells after treated with different formulations were investigated by western-blot analysis (**Figure 5B**). In contrast to the control group, the HSP70 and HSP90 expression were significantly elevated in HepG-2 cells after treated with PDA@hm+NIR, validating that the generation of HSPs could be triggered by increasing the temperature of cancer cells after PTT treatment. However, the expression of HSP70 and HSP90 in cells after thermal treatment could be significantly reduced by PDA@hm@GOx and PDA@hm@CQ@GOx. These results demonstrated that the interference of glucose metabolism by GOx could effectively suppress the HSPs expression during thermal therapy.

According to previous reports, the induction of autophagy in cancer cells would occur in response to chemotherapy, metabolic stress and thermal treatment 31,51-53. Hence, targeting autophagy has been recognized as complementary strategy in cancer therapy due to the resistance of autophagy-related tolerance. Next, we investigated the intracellular autophagy situations under different treatments. So far, TEM and western-blot analysis were the most reliable methods to evaluate autophagy level in cells. Therefore, we investigated the autophagy behaviors in cancer cells after different treatments via both western-blot and TEM analysis. During autophagy process, microtubule-associated light chain 3-I (LC3-I) protein is recruited and accumulated on the membrane of the autophagosomes, and subsequently carries out the transition process from LC3 I form into LC3 II form, which is the mark of the autophagosomes maturation 54. According to **Figure 5C**, both GOx and PDA@hm+NIR-treated cells displayed increased LC3-II protein level compared with the control cells, confirming the activation of autophagy by starvation and photothermal treatment. CQ-treated cells also exhibited an obvious increased LC3-II protein as it inhibited the degradation of the autophagosomes via disturbing the lysosome activity and thus caused LC3-II protein accumulation within the cytoplasm 55. Specially, significant increased expression of LC3-II protein could be seen from PDA@hm@CQ@GOx+NIR-treated cells. Furthermore, TEM analysis was utilized to directly observe the autophagy behavior of HepG-2 cells after different treatments. As shown in **Figure 5D**, in consistence with the western-blot results, both GOx and PDA@hm+NIR-treated cells contained more autophagosomes and other autophagic vesicles as compared to the control cells. Due to the inhibition effect of the degradation of autolysosomes, many autophagic vesicles could be clearly observed after CQ treatment. Meanwhile, much more autophagic vesicles could be seen from PDA@hm@CQ@GOx+NIR-treated cells. Altogether, these results strongly confirmed that PDA@hm@CQ@GOx+NIR-treated cells could induce autophagy inhibition in cancer cells, sensitizing the PDA@hm-mediated PTT and GOx-mediated starvation therapeutic effect. With the western-blot results, the outstanding therapy effects of PDA@hm@CQ@GOx+NIR treatment could simultaneously be attributed to the sensitivity of tumor cells to hyperthermia via GOx-mediated starvation and CQ-mediated autophagy inhibition effect.

### *In vivo* imaging and anti-tumor studies

**Figure 6:**
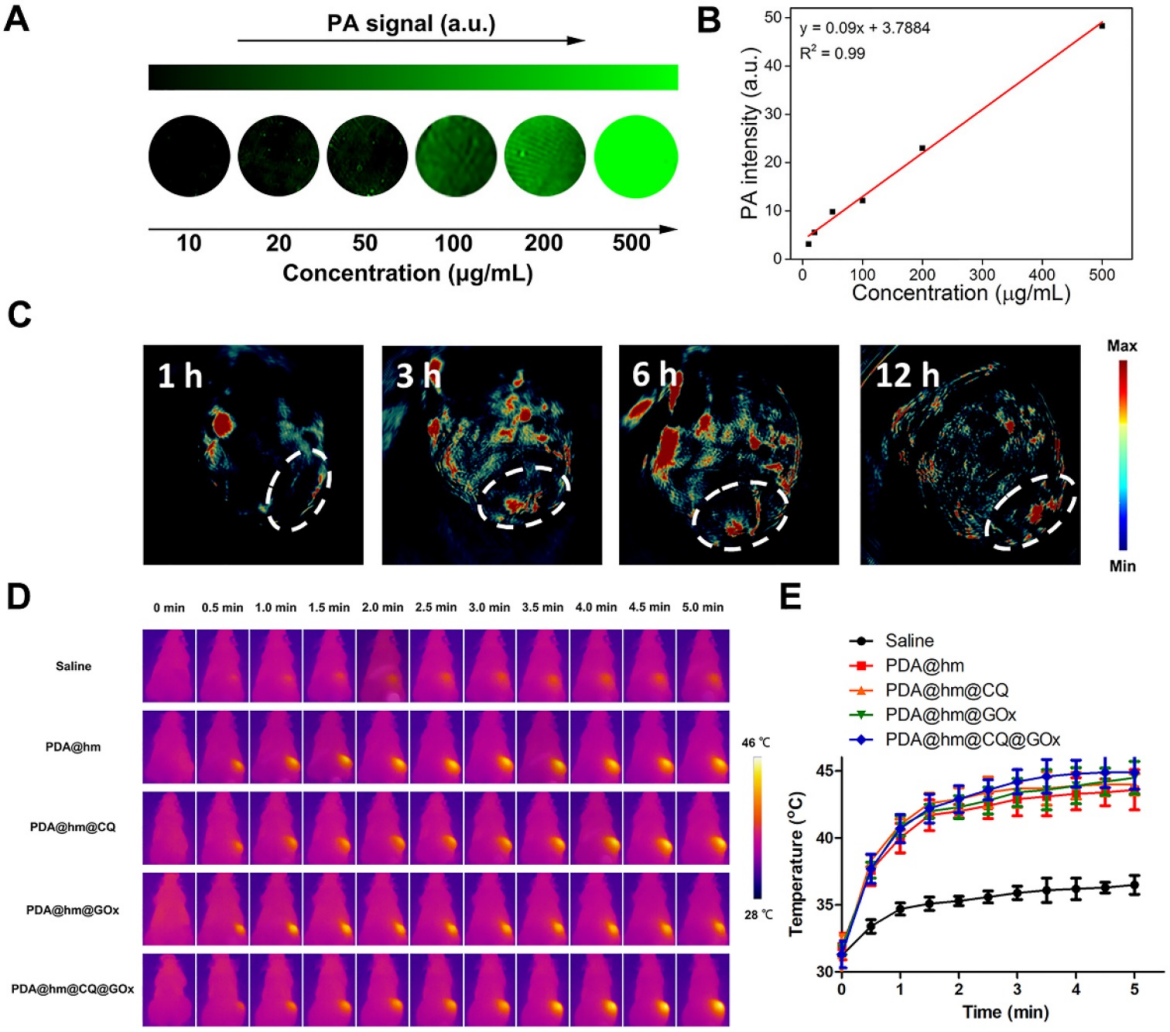
** (A)** *In vitro* PA imaging of the PDA@hm@CQ@GOx solution at different concentrations. **(B)** The corresponding quantitative curve of PA intensity at different concentrations. **(C)** *In vivo* PA images of mice taken at 1 h, 3 h, 6 h and 12 h after i.v., injection of PDA@hm@CQ@GOx. **(D)** IR thermal images of HepG-2 tumor-bearing mice exposed to 808 nm laser for 5 min. **(E)** The corresponding temperature changes at the tumor sites.

It has been illustrated that the PDA component could be served as a contrast agent for photoacoustic (PA) imaging. Therefore, the possibility of PDA@hm@CQ@GOx for PA imaging was firstly assessed *in vitro*. As depicted in **Figure 6A** and **6B**, the PA signal heightened with the increasing concentrations of PDA@hm@CQ@GOx and exhibited a linear relationship between the PA signal intensity and concentration. The results demonstrated the potential PA imaging capability of PDA@hm@CQ@GOx. In addition, the blood hemolysis of PDA@hm@CQ@GOx was carried out and the result demonstrated that the favorable blood compatibility of these nanoparticles (**Figure S10**). Subsequently, in vivo PA imaging was carried out on HepG-2 tumor-bearing mice after intravenous injection with PDA@hm@CQ@GOx. Remarkably, the PA signals in the tumor site could be clearly distinguished and observed in PDA@hm@CQ@GOx-injected mice after 12 h (**Figure 6C**). Collectively, the *in vitro* and *in vivo* imaging results indicated that the PDA@hm@CQ@GOx nanoparticles could be used for tumor PA imaging to a certain degree.

**Figure 7:**
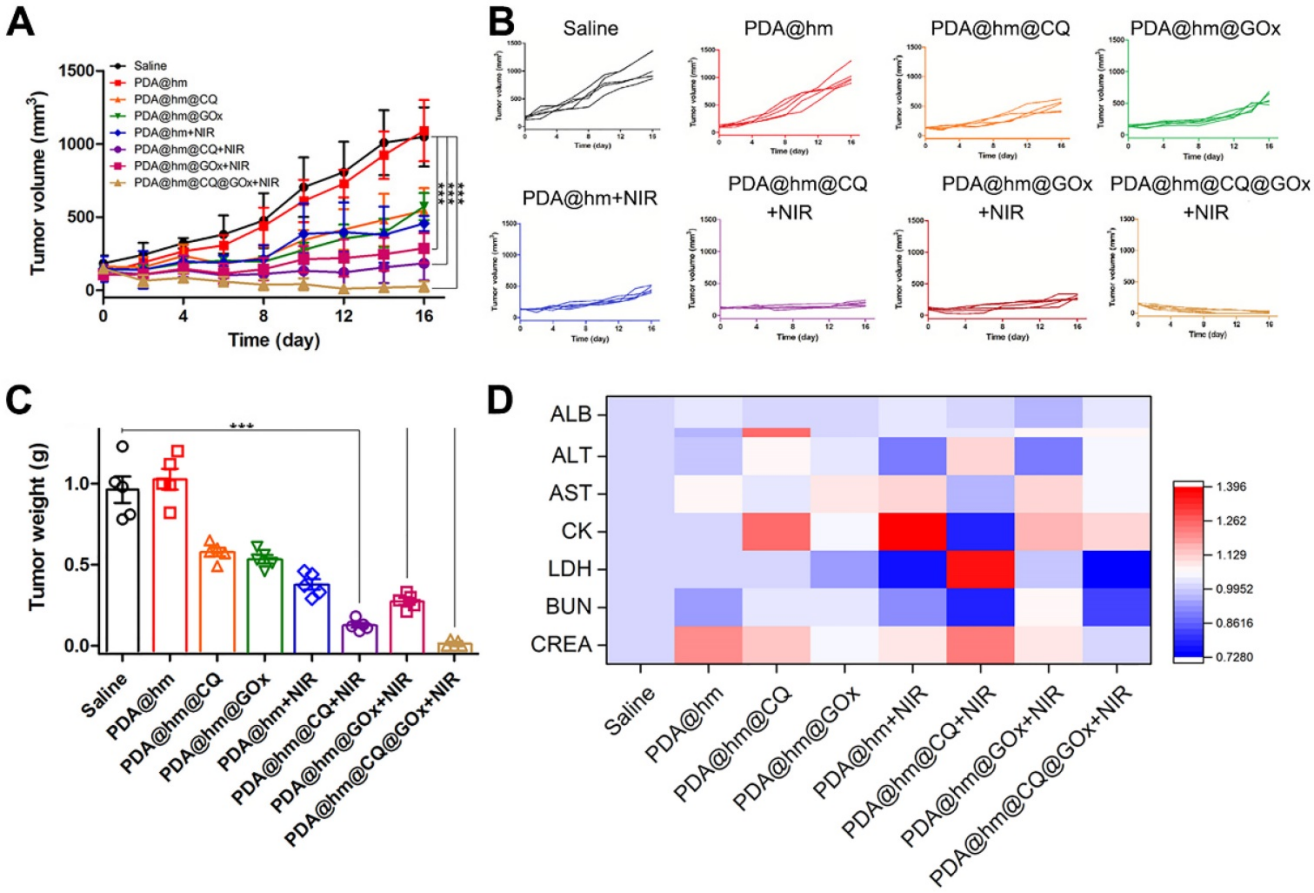
**(A)** Tumor growth curves of mice after various treatments in 16 days. **(B)** Individual tumor growth curves of the mice. **(C)** The average tumor weights after various treatments for 16 days. **(D)** Blood analysis of mice at 16 days after various treatments with heat map compared with the saline-treated mice. ***p < 0.001 versus the control group.

Encouraged by the *in vitro* synergistic therapeutic results, we then systematically investigated the *in vivo* antitumor effects of different formulations with or without laser treatment in HepG-2 tumor-bearing mice. When the tumor volume reached to ~100 mm^3^, the mice were randomly divided into 8 groups and then intravenously injected with saline, PDA@hm, PDA@hm@CQ, PDA@hm@GOx, PDA@hm+NIR, PDA@hm@CQ+NIR, PDA@hm@GOx+NIR and PDA@hm@CQ@GOx+NIR, respectively. An IR thermal camera was used to record the tumor temperature changes under NIR laser irradiation. As illustrated in **Figure 6D** and **6E**, the photothermal heating profile displayed similar temperature increasement at the tumor regions in all the PDA-containing nanoparticles-treated groups and the ultimate tumor temperatures reached to about 45 °C. In comparison, the temperature in saline-injected mice only exhibited a slight increasement under the same laser irradiation. The tumor volumes were continuously measured and recorded after various treatments and the progressive growth curves of the implanted tumors were summarized in **Figure 7A** and **7B**. It demonstrated that the tumors in saline- and PDA@hm-treated mice grew rapidly. In the absence of NIR laser irradiation, both PDA@hm@GOx- and PDA@hm@CQ-treated mice displayed a moderate inhibition effect in tumor growth, demonstrating that individual GOx- and CQ-incorporated nanoparticles could inhibit tumor growth *in vivo*. PDA@hm+NIR-treated group only showed partially delay on the tumor growth, which could be attributed to that the low-temperature thermal (about 45 °C) was not enough for effective tumor ablation. Interestingly, the mice treated with PDA@hm@GOx or PDA@hm@CQ under NIR laser irradiation showed higher inhibition effects than any other single treatment on the tumor growth, implying the enhanced therapeutic effects of GOx and CQ supplemented in the low-temperature PTT. Extraordinary tumor regression could be observed in PDA@hm@CQ@GOx+NIR-treated mice and no relapse occurred at the end of the experimental period. The photographs of typical mice received different treatments at 16 days were also recorded (**Figure S11**). Such a superior antitumor effect resulted from enhanced low-temperature PTT effects through synchronously regulating glucose metabolism and inhibiting autophagy. All mice were euthanized on the 16th day and the tumor tissues from each group were collected and weighted. As shown in **Figure 7C**, the observed trends of average tumor weights also confirmed that the superior anti-tumor performance of the PDA@hm@CQ@GOx+NIR treatment. These results suggested that low-temperature PTT supplemented with GOx-mediated starvation and CQ-mediated autophagy inhibition could result in more efficient therapeutic effects.

During the therapeutic period, the body weights of mice were also measured and recorded. As shown in **Figure S12**, no obvious body weight drop was seen from all mice, implying that all treatments had negligible acute systemic toxicity. In addition, various serum biochemistry indexes, including albumin (ALB), alkaline phosphatase (ALP), alanine aminotransferase (ALT), aspartate transaminase (AST), creatine kinase (CK), lactate dehydrogenase (LDH), blood urea nitrogen (BUN), and creatinine (CREA), had no significant changes (**Figure 7D**). These results also demonstrated the good biocompatibility of PDA@hm@CQ@GOx with or without laser treatment. Moreover, a typical hematoxylin and eosin (H&E) staining method was performed to analyze the corresponding histological changes of the major organs (heart, liver, spleen, lung, kidney) after different treatments. No evident physiological morphology changes were observed in all organs, demonstrating no obvious systemic toxicity of PDA@hm@CQ@GOx (**Figure S13**). Taken together, the above results demonstrated that PDA@hm@CQ@GOx had a good biocompatibility *in vivo*.

## Conclusions

In conclusion, we successfully developed rattle-structured polydopamine core/hollow mesoporous silica shell nanoparticles (PDA@hm). A strategy of augmented low-temperature PTT in combination with cells autophagy inhibition and energy metabolism regulation was proposed for trimodal complementary cancer therapy. The glucose consumer GOx and autophagy inhibitor CQ were decorated onto the surface and loaded into the cavity of PDA@hm, respectively. The GOx-mediated tumor starvation would directly suppress the expression of HSPs, resulting in an enhanced low-temperature PTT induced by PDA nanocore. Excellently, inhibition of autophagy by the released CQ made up for the loss of low-temperature PTT and starvation efficiencies by PTT- and starvation-activated autophagy, realizing augmented therapeutic efficacy. The improved therapeutic efficacy from the mild thermal combination therapy was confirmed both *in vitro* and *in vivo*. In addition, the PDA@hm@CQ@GOx could be used as a PA contrast agent for PA imaging. Overall, this study provided a proof-of-concept for enhanced mild PTT through regulating glucose metabolism and inhibiting autophagy.

## Supplementary Material

Supplementary figures.

## Abbreviations

PA: photoacoustic
PTT: photothermal therapy
GOx: glucose oxidase
PDA: polydopamine
CQ: chloroquine
FDA: Food and Drug Administration
HSP: heat shock protein
PDA@hm: polydopamine core/hollow mesoporous silica shell nanoparticles
CCK-8: cell counting kit-8
ICG: indocyanine green
TEOS: tetraethyl orthosilicate
CTAB: hexadecyl trimethyl ammonium bromide
PVP: polyvinylpyrrolidone
APTES: 3-aminopropyltrimethoxysilane
TEM: transmission electron microscopy
SEM: scanning electron microscopy
DLS: dynamic light scanning
CLSM: confocal laser scan microscopy
PVDF: poly (vinylidene difluoride)
MSOT: multispectral optoacoustic tomography scanner
SD: standard deviation
ANOVA: one-way analysis of variance
BET: brunauer-emmett-teller
NIR: near-infrared
FITC: fluorescein isothiocyanate
PI: propidium iodide
LC3: microtubule-associated light chain 3
ALB: albumin
ALP: alkaline phosphatase
ALT: alanine aminotransferase
AST: aspartate transaminase
CK: creatine kinase
LDH: lactate dehydrogenase
BUN: blood urea nitrogen
CREA: creatinine
