# Supplementary material for: Complementary autophagy inhibition and glucose metabolism with rattle-structured polydopamine@mesoporous silica nanoparticles for augmented low-temperature photothermal therapy and in vivo photoacoustic imaging
Source: Theranostics. 2020 Jun 5;10(16):7273–86. doi: 10.7150/thno.44668 (PMC7330850; doi:10.7150/thno.44668)
Supplement: Supplementary file 1 — Supplementary figures. [file thnov10p7273s1.pdf]

Supporting Information

Complementary autophagy inhibition and glucose metabolism with rattle-structured polydopamine@mesoporous silica nanoparticles for augmented low-temperature photothermal therapy and *in vivo* photoacoustic imaging

Leihou Shao [1,3*], Yunhao Li [2*], Feifei Huang [4], Xuan Wang [1,5], Jianqing Lu [1], Fan Jia [1,5], Zian Pan [1,5], Xinyue Cui [1], Guanglu Ge [1,5], Xiongwei Deng [1], and Yan Wu [1,5]

1. CAS Key Laboratory for Biomedical Effects of Nanomaterials and Nanosafety, CAS Key Laboratory of Standardization and Measurement for Nanotechnology, CAS Center for Excellence in Nanoscience, National Center for Nanoscience and Technology, Beijing 100190, P. R. China
2. Department of General Surgery, Peking Union Medical College Hospital, Peking Union Medical College, Chinese Academy of Medical Sciences, Beijing 100730, P. R. China
3. Beijing Key Laboratory of Organic Materials Testing Technology and Quality Evaluation, Beijing Center for Physical and Chemical Analysis, Beijing, 100089, China
4. The Key Laboratory of Beijing City on Preparation and Processing of Novel Polymer Materials, Beijing University of Chemical Technology, Beijing, 100029, China
5. University of Chinese Academy of Sciences, Beijing 100049, P. R. China
* These authors contributed equally to this work.

Corresponding author: dengxw2018@nanoctr.cn (X. Deng), wuy@nanoctr.cn (Y. Wu).

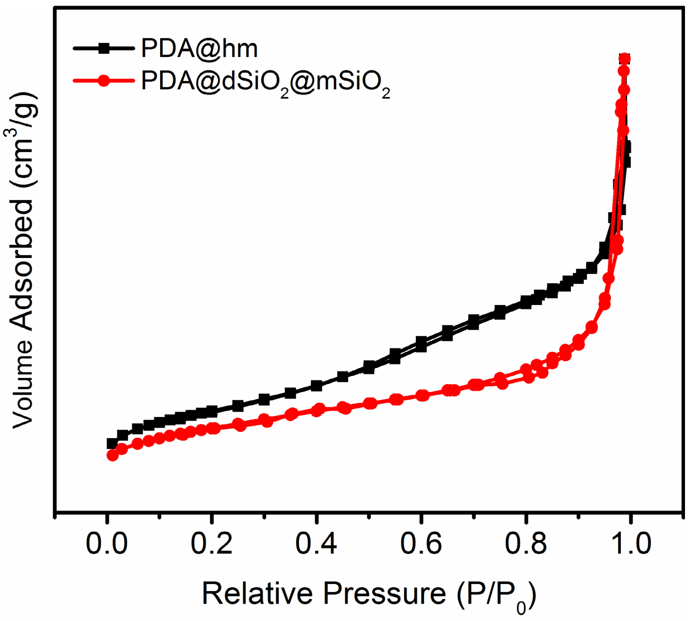

Figure S1. N_2 adsorption-desorption isotherms of PDA@hm.

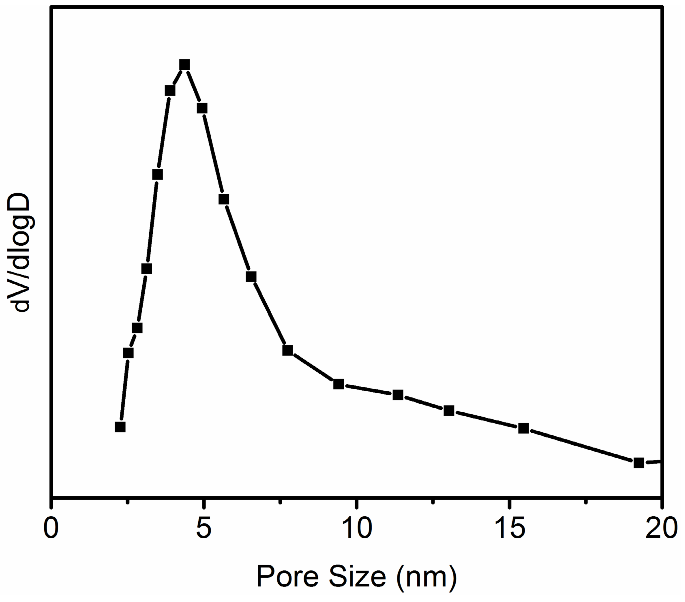

Figure S2. The average pore diameter of PDA@hm.

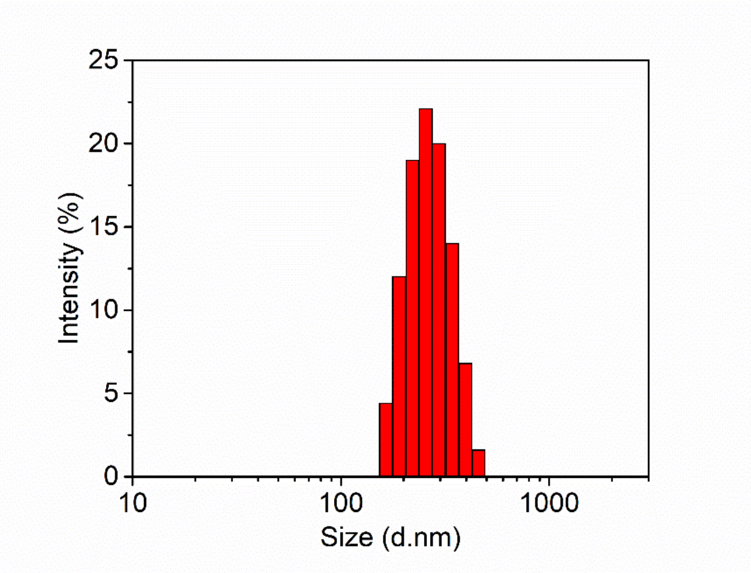

Figure S3. DLS analysis of PDA@hm@CQ@GOx.

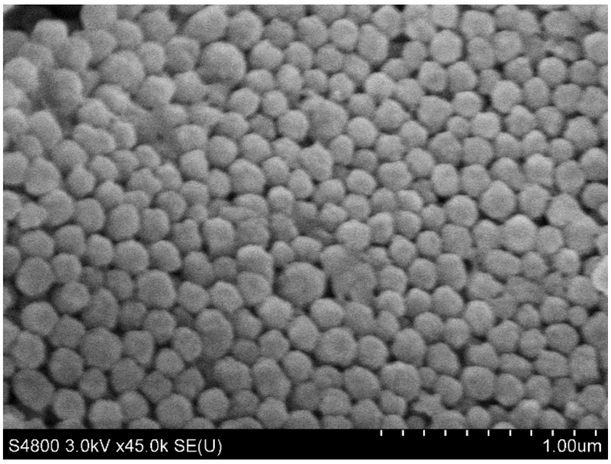

Figure S4. SEM image of PDA@hm@CQ@GOx.

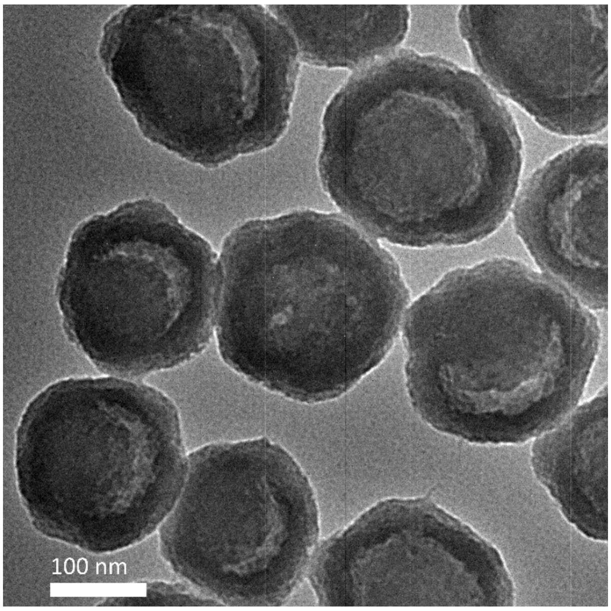

Figure S5. TEM image of PDA@hm@CQ@GOx.

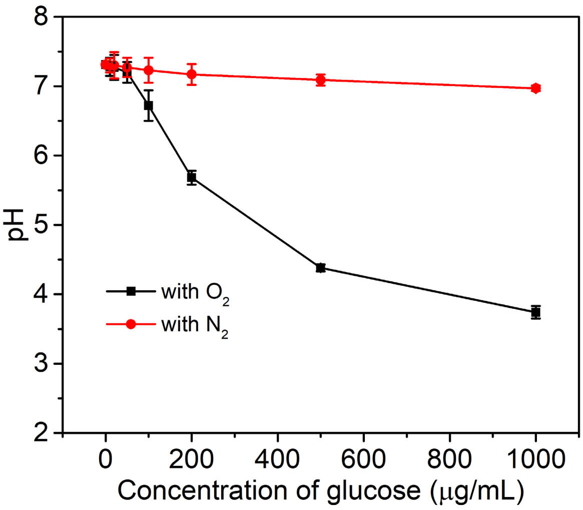

Figure S6. The pH value changes arising from the reaction between PDA@hm@CQ@GOx and glucose in an O_2/N_2 condition.

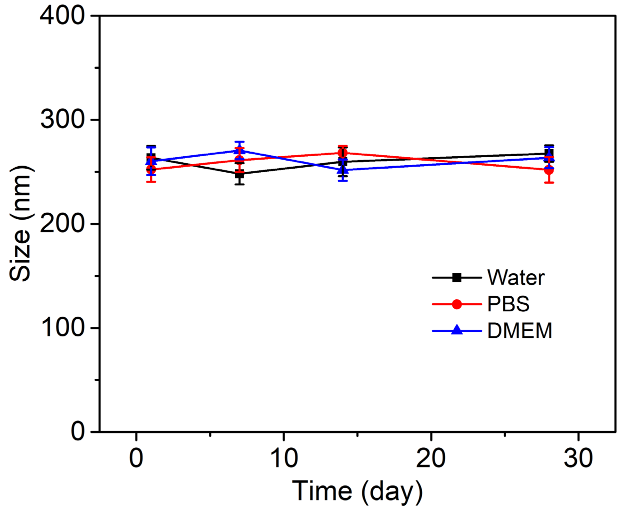

Figure S7. Size changes of PDA@hm@CQ@GOx dispersions in water, PBS, and cell culture medium containing 10% FBS at different time points.

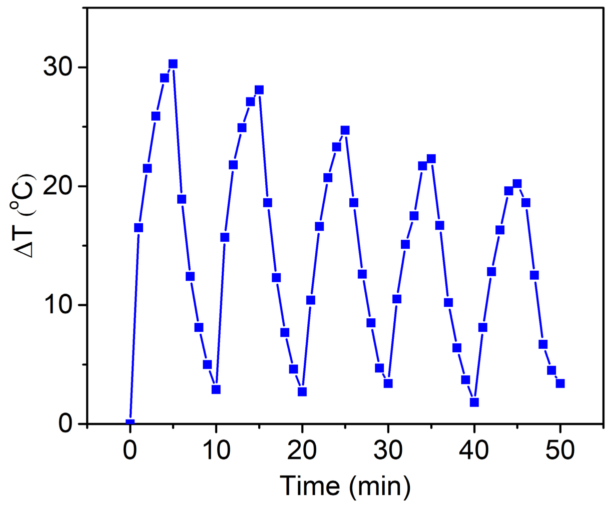

Figure S8. Temperature changes of the ICG solution during the five laser on/off cycles.

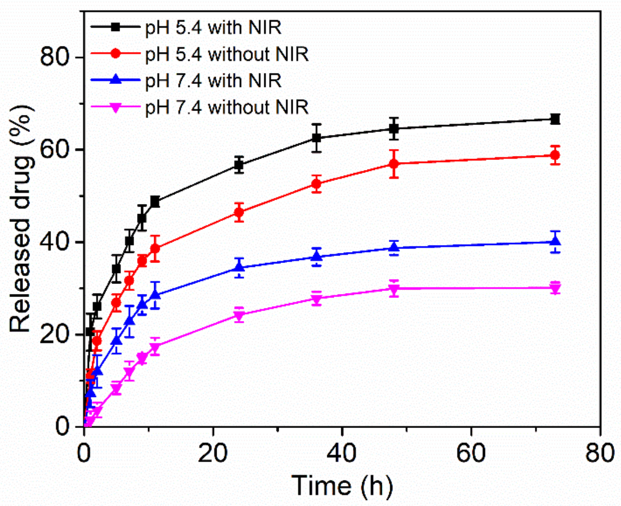

Figure S9. Release profiles of CQ from PDA@hm@CQ@GOx at different pH values with or without NIR laser irradiation.

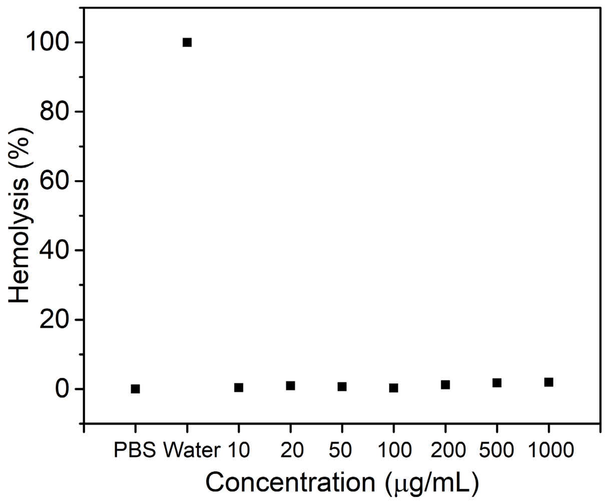

Figure S10. Hemolysis assay with different concentrations of PDA@hm@CQ@GOx (negative control: PBS; positive control: water).

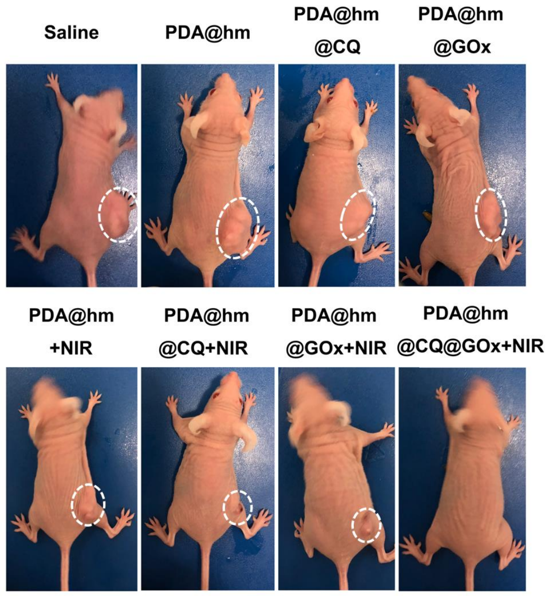

Figure S11. Representative images of the tumor-bearing mice from each group before euthanizing the animal on day 16.

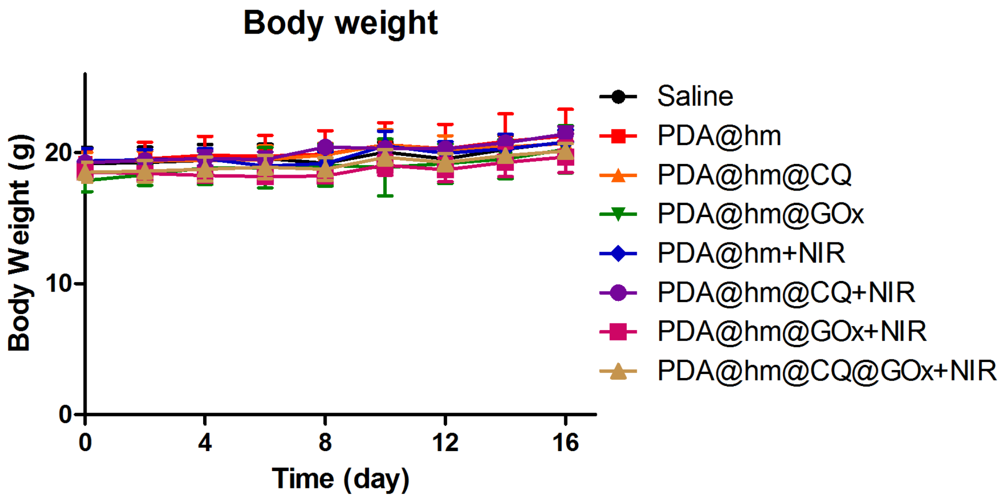

Figure S12. Relative body weights measured from all groups of mice.

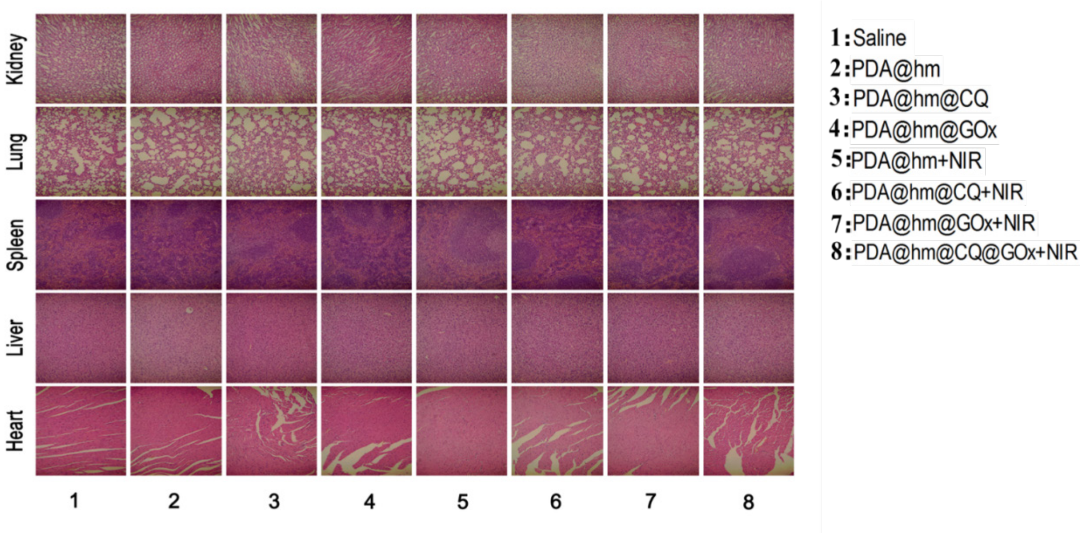

Figure S13. Histological analysis of the major organs (heart, liver, spleen, lung, kidney) after different treatments.
